# School readiness in children with early-onset chronic liver disease: a population-based linked data cohort in England

**DOI:** 10.1136/archdischild-2025-329409

**Published:** 2026-02-16

**Authors:** Nicolás R Libuy, Marianne Samyn, Katie L Harron

**Affiliations:** 1 School of Government, Universidad Adolfo Ibanez, Santiago, Chile; 2 Centre for Longitudinal Studies, University College London Social Research Institute, London, UK; 3 Paediatric Liver Centre, King’s College Hospital NHS Foundation Trust, King’s College Hospital Charity, London, UK; 4 Great Ormond Street Institute of Child Health, University College London, London, UK

**Keywords:** Paediatrics, Epidemiology, Child Development, Child Health

## Abstract

**Background:**

Chronic liver disease (CLD) affects 2–10 per 10 000 children. While prior evidence links CLD to lower cognitive ability, its impact on school readiness remains unclear.

**Objective:**

To study the association between early-onset chronic liver disease (CLD; <5 years) and school readiness at age 5.

**Design:**

A population-based cohort study using linked health and education data from the Education and Child Health Insights from Linked Data project in England. Children born between 2002 and 2012 with teacher assessments at age 4–5 years (2007/2008–2016/2017) were included. CLD diagnoses were identified using the International Statistical Classification of Diseases and Related Health Problems, 10th revision and Operating Procedure Codes Supplement 4 hospital codes. Multivariable regression assessed standardised developmental outcomes in up to seven domains.

**Results:**

Of 5 084 671 pupils, 0.1% (n=3672) were diagnosed with early-onset CLD by age 5. Of children in 2011/2012–2016/2017 with CLD, 55.8% did not achieve a good level of development, compared with 35.3% without CLD (adjusted relative risk: 1.40, 95% CI 1.34 to 1.46). Standardised total point scores were 0.69 lower (95% CI 0.58 to 0.81) in 2007/2008–2010/2011 and 0.41 lower (95% CI 0.36 to 0.46) in 2011/2012–2016/2017. Children with CLD performed worse than those without CLD in all areas of development, with the strongest effect sizes in the physical development domain. Larger effects were observed among girls, those with more severe disease and those from less deprived areas.

**Conclusions:**

Children with early-onset CLD face greater cognitive risk than their peers affected by liver transplantation hospital stays. Further exploration of longitudinal educational outcomes and their relationship with specific liver disease-related factors is needed.

**WHAT IS ALREADY KNOWN ON THIS TOPIC**

- Children with chronic liver disease (CLD) may experience neurodevelopmental difficulties, particularly those undergoing liver transplantation. However, there is limited large-scale evidence on how early-onset CLD affects child development and school readiness.

**WHAT THIS STUDY ADDS**

- This is the first population-based study to assess school readiness in children with early-onset CLD using linked health and education records. Children with CLD scored lower across all developmental domains, with the greatest deficits observed in physical development. Effects were more pronounced in girls, children with severe disease and those from less deprived areas.

**HOW THIS STUDY MIGHT AFFECT RESEARCH, PRACTICE OR POLICY**

- Findings support integrating neurodevelopmental assessments into routine care for children with CLD and improving coordination between health and education services. Early identification and tailored educational interventions may help mitigate long-term disadvantages in this vulnerable group.

## Introduction

 Liver disease in children is rare, and its prevalence and incidence are not well defined. Whereas jaundice initially after birth is not uncommon and not necessarily pathological, delayed clearance of jaundice or obstructive jaundice suggests an intrinsic liver problem, which is estimated to occur in 1/2500 live births.^1^ The most common condition presenting in infancy is biliary atresia (BA), with an estimated prevalence of 1/5000–20 000 live births depending on geographic area.^2^ When the liver disease progresses and becomes irreversible or when presenting acutely, liver transplantation can be indicated. Paediatric liver transplant recipients make up 11% of all liver transplants in the UK. Long-term survival is excellent, particularly for children undergoing transplant before the age of 9 years who have an estimated 30-year patient survival of 74%.^3^

Despite this, children with chronic liver disease (CLD), including those who have undergone liver transplantation, are at increased risk of neurodevelopmental difficulties in all domains, including cognitive, behavioural and motor.^4^ Up to 42% of children following liver transplant require additional educational support. However, the current literature relies on single-centre studies with a high diversity in used test batteries and questionnaires and population-based outcomes, including school performance, are lacking.^4^

In order to generate more robust evidence on the development of children with CLD, we used a population-based administrative data cohort of 5 million pupils in England to evaluate the association between early-onset CLD (diagnosed by age 5; referred to from now as CLD for brevity) and indicators of school readiness across a range of developmental areas at age 5.

## Methods

### Study population

We used the Education and Child Health Insights from Linked Data (ECHILD) database, which links several administrative datasets from Hospital Episode Statistics (HES) and the National Pupil Database (NPD) in England.^5^,^7^ ECHILD includes information about pupil demographics and educational outcomes. It also includes longitudinal information about hospital admissions, accident and emergency attendances, outpatient appointments and deaths.^5^ The datasets were linked by National Health Service (NHS) England, and the linkage process has been described in detail elsewhere.^8^ Our study population included all pupils captured in the education data at age 4/5 in the Early Years Foundation Stage (EYFSP), between academic years 2007/2008 and 2016/2017. This cohort was born between September 2002 and August 2012. We restricted our population to children who were born in NHS hospitals in England and who could be linked to their birth episode in HES, so that we had a complete diagnostic history on all of our study population. This allowed us to be confident in our classification of disease status. Those who were born outside of an NHS hospital in England, or whose birth record could not be linked due to missing data on matching fields, were therefore not included (as we would not have known if they had a previous diagnosis or not). We excluded babies recorded as having a gestational age <24 weeks (~0.02% of the cohort; online supplemental figure S1).

### Exposure and outcome

Children with any diagnosis of CLD in a hospital record by age 5 were classified as having early-onset CLD, using an International Statistical Classification of Diseases and Related Health Problems, 10th revision (ICD-10) diagnostic codes list and (online supplemental table S1). The code list was developed using primary diagnosis codes for patients admitted after 1 January 2017 under paediatric liver specialty at King’s College Hospital, London, and modified in consultation with clinical coders and clinicians to capture the aetiology of paediatric CLD.^9^ Children who only presented with jaundice before 12 months but no other CLD pathology by age 5 were not classified as having CLD, since unconjugated jaundice is a relatively prevalent condition among infants not associated with CLD later in life.

We classified the severity of CLD according to whether the child was recorded as having a liver transplant by age 5 using ICD-10 codes and Operating Procedure Codes Supplement 4 (OPCS-4) codes (online supplemental tables S1 and S2). We also classified severity according to the number of days children spent in hospital with a CLD-related diagnosis (grouped into quartiles).

Pupil attainment was identified from official teacher assessments of children’s development at the end of the Early Years Foundation Stage, at age 5. Attainment captures the summative assessment of early learning goals across up to seven areas of learning. A binary outcome indicating whether pupils achieved a ‘good level of development’ as used to assess the development of children evaluated in the academic years 2011/2012–2016/2017. A binary outcome was not available in NPD for academic years 2007/2008–2010/2011. Instead, child development was evaluated using the standardised total point score in three developmental areas for academic years 2007/2008–2010/2011 (personal, social and emotional; communication, language and literacy; problem solving, reasoning and numeracy) and seven developmental areas for academic years 2011/2012–2016/2017 (communication and language; physical; personal, social and emotional; literacy; understanding the world; mathematics; expressive arts, designing and making).

### Covariates

ECHILD includes a series of clinical and sociodemographic factors potentially associated with CLD and school attainment. Clinical factors include sex, gestational age (in completed weeks, grouped as 24–31, 32–33, 34, 36, 37–38, 39, 40 and 41–43), size for gestation (small 10th percentile, normal, large >90th percentile) and maternal age (<20, 20–24, 25–30, 30–34, 35–39 and 40–45 years). Socioeconomic factors include ethnicity (white, Asian, black, Chinese, mixed and other, derived from NPD and supplemented by HES where missing) and quartile of the Income Deprivation Affecting Children Index (IDACI; an area-based indicator of socioeconomic status in the areas where pupils were living during the EYFSP). We also included the academic years in which attainment was recorded (2011/2012, 2012/2013, 2013/2014, 2014/2015 and 2016/2017) to control for differences across academic cohorts. We included the year-month of birth (September 2002–August 2012) as the month of birth has been shown to be strongly associated with attainment at age 5.^10^

### Statistical analysis

We used Poisson models to estimate the adjusted relative risk (RR) of not achieving a good level of development for children with CLD by age 5 compared with those with no diagnosis of CLD. To determine the association between CLD and standardised scores in different areas of development, we used linear models (11 different models, covering total scores and each domain separately for the two academic periods).^11 12^ Our fully adjusted models included sex, ethnicity, IDACI quartile, gestational age (in weeks), size for gestation, maternal age, academic year and month of birth. We evaluated results with multiple imputation and complete-case analysis. Given similar results from complete-case and multiple-imputation analyses and because missingness likely depends on observed covariates, we report adjusted estimates from complete-case analyses only (table 1).^13^

**Table 1:** Study population characteristics according to diagnosis of chronic liver disease by age 5

Characteristic	Chronic liver disease by age 5 No. (%)	
	No	Yes
Total	5 080 999 (99.9)	3672 (0.1)
Sex		
Male	2 470 270 (48.6)	1676 (45.6)
Female	2 610 729 (51.4)	1996 (54.4)
Ethnicity		
White	3 931 332 (77.4)	2613 (71.2)
Asian	496 030 (9.8)	555 (15.1)
Black	254 618 (5.0)	204 (5.6)
Chinese	19 823 (0.4)	<30 (0.0)
Other	68 893 (1.4)	67 (1.8)
Mixed	297 223 (5.8)	206 (5.6)
Missing	13 080 (0.3)	<10 (0.0)
Income Deprivation Affecting Children Index score (quartile)		
First (less deprived)	1 169 357 (23.0)	715 (19.5)
Second	1 168 182 (23.0)	810 (22.1)
Third	1 165 063 (22.9)	895 (24.4)
Fourth (more deprived)	1 167 265 (23.0)	1025 (27.9)
Missing	411 132 (8.1)	227 (6.2)
Gestational age (weeks)		
<32	33 268 (0.7)	259 (7.1)
32–33	31 349 (0.6)	75 (2.0)
34–36	177 792 (3.5)	253 (6.9)
37–38	725 908 (14.3)	604 (16.4)
39	862 340 (17.0)	517 (14.1)
40	1 109 827 (21.8)	593 (16.1)
41+	938 236 (18.5)	400 (10.9)
Missing	1 202 279 (23.7)	971 (26.4)
Size for gestation		
Small (<10th centile)	343 210 (6.8)	449 (12.2)
Normal	3 080 337 (60.6)	1940 (52.8)
Large (>90th centile)	379 719 (7.5)	240 (6.5)
Missing	1 277 733 (25.1)	1043 (28.4)
Maternal age (years)		
<20	315 280 (6.2)	242 (6.6)
20–24	951 717 (18.7)	731 (19.9)
25–30	1 311 278 (25.8)	919 (25.0)
30–34	1 366 802 (26.9)	878 (23.9)
35–39	761 514 (15.0)	481 (13.1)
40–45	168 240 (3.3)	137 (3.7)
Missing	206 168 (4.1)	284 (7.7)

Small cell sizes have been suppressed.

To investigate whether outcomes differed by severity of disease, we fitted Poisson models with categories of liver disease (transplant vs no transplant; length of hospital stay). To investigate whether the effect of CLD on educational outcomes varied according to sex, preterm birth or deprivation, we included interaction terms for these variables within the Poisson model.

Analyses were conducted in Stata V.8.0 using robust (sandwich) variance estimators for all Poisson and linear models. For Poisson models, we assessed overdispersion by comparing the mean and variance of the outcome and inspecting deviance and Pearson residuals. For linear models, residual plots were reviewed for normality and homoscedasticity. No substantial violations of model assumptions were detected.

## Results

Of 5 084 671 pupils included in our sample (online supplemental figure S1), 0.1% (n=3672) were defined as having early-onset CLD (table 1). Of these, 9% (n=331) (or 1:15 361 children overall) had a diagnosis of BA; 1.4% (n=53) had a diagnosis of autoimmune liver disease, and 7.2% (n=264) had a liver transplant. The majority (58%) of children were diagnosed in the first year of life (online supplemental table S3).

Girls, children living in more deprived areas and children born at lower gestational ages were over-represented among the CLD group (table 1).

**Figure 1:**
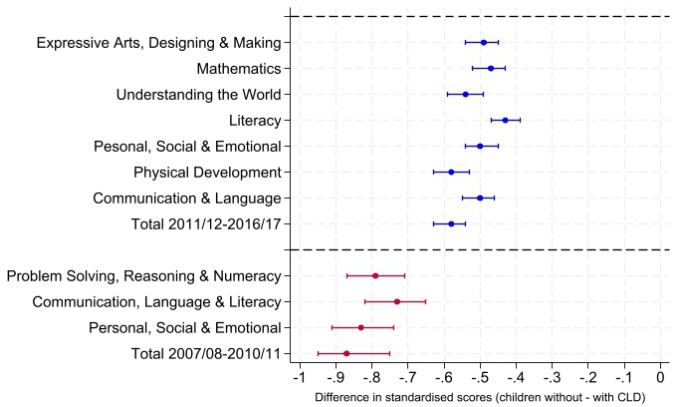
Difference (and 95% CIs) in standardised scores in the Early Years Foundation Stage Profile at age 5, comparing children with and without chronic liver disease by age 5, for each developmental area. Red=2007/2008–2010/2011; Blue=2011/2012–2016/2017.

In the cohort 2011/2012–2016/2017, 55.8% of children with CLD did not achieve a good level of development, compared with 35.3% of those without CLD (table 2). Compared with children without CLD, children with CLD scored 0.69 lower (95% CI 0.58 to 0.81) in cohort 2007/2008–2010/2011 and 0.41 lower (95% CI 0.36 to 0.46) in 2011/2012–2016/2017 (figure 1, online supplemental table S4). Children with CLD performed worse than those without CLD in all areas of development, and effect sizes were strongest for the physical development domain (figure 1 and online supplemental table S5).

**Table 2:** Adjusted relative risk of not achieving a good level of development comparing children with and without chronic liver disease by age 5 (academic years 2011/2012–2016/2017)

Characteristic	Good level of development No. (%)		Adjusted relative risk (95% CI)
	Did achieve	Did not achieve	
Total	1 781 041 (64.7)	973 358 (35.3)	
Chronic liver disease by age 5	993 (44.2)	1256 (55.8)	1.40 (1.34 to 1.46)
Sex			
Female	971 656 (72.6)	367 359 (27.4)	Ref.
Male	809 385 (57.2)	605 999 (42.8)	1.58 (1.57 to 1.58)
Ethnicity			
White	1 376 851 (65.2)	735 189 (34.8)	Ref.
Asian	169 794 (61.8)	105 093 (38.2)	1.00 (0.99 to 1.00)
Black	87 722 (62.9)	51 756 (37.1)	0.95 (0.94 to 0.95)
Chinese	8014 (64.7)	4365 (35.3)	1.01 (0.98 to 1.03)
Any other ethnic group	23 401 (58.2)	16 833 (41.8)	1.09 (1.08 to 1.10)
Mixed	112 865 (65.8)	58 689 (34.2)	0.92 (0.91 to 0.92)
Income Deprivation Affecting Children Index score			
First (less deprived)	485 402 (72.7)	182 113 (27.3)	Ref.
Second	475 936 (67.8)	225 991 (32.2)	1.16 (1.15 to 1.16)
Third	451 361 (62.0)	276 173 (38.0)	1.33 (1.32 to 1.33)
Fourth (more deprived)	364 020 (56.0)	286 034 (44.0)	1.46 (1.45 to 1.46)
Gestational age (weeks)			
24–31	8272 (41.9)	11 472 (58.1)	1.67 (1.64 to 1.68)
32–33	9536 (51.7)	8912 (48.3)	1.42 (1.40 to 1.44)
34–36	60 562 (57.3)	45 108 (42.7)	1.26 (1.25 to 1.27)
37–38	268 647 (62.0)	164 536 (38.0)	1.15 (1.14 to 1.15)
39	346 728 (65.3)	184 269 (34.7)	1.06 (1.05 to 1.06)
40	452 957 (66.9)	224 099 (33.1)	Ref.
41–43	386 373 (67.6)	185 214 (32.4)	0.98 (0.97 to 0.98)
Size for gestation			
Small (<10 centile)	116 479 (57.4)	86 292 (42.6)	1.19 (1.18 to 1.19)
Normal	1 239 210 (65.5)	653 408 (34.5)	Ref.
Large (>90 centile)	159 287 (68.4)	73 424 (31.6)	0.95 (0.94 to 0.95)
Maternal age (years)			
<20	80 529 (51.2)	76 765 (48.8)	1.48 (1.47 to 1.49)
20–24	299 434 (57.5)	221 207 (42.5)	1.32 (1.31 to 1.32)
25–30	478 662 (64.5)	263 357 (35.5)	1.14 (1.13 to 1.14)
30–34	514 550 (70.1)	219 329 (29.9)	Ref.
35–39	288 207 (69.4)	126 868 (30.6)	1.02 (1.01 to 1.02)
40–65	65 103 (66.5)	32 781 (33.5)	1.12 (1.10 to 1.12)

The crude RR of not achieving a good level of development comparing children with CLD to those without was 1.60 (95% CI 1.55 to 1.65). The effect size was somewhat attenuated after accounting for sociodemographic and clinical factors: (1.40, 95% CI 1.34 to 1.46; table 2).

**Figure 2:**
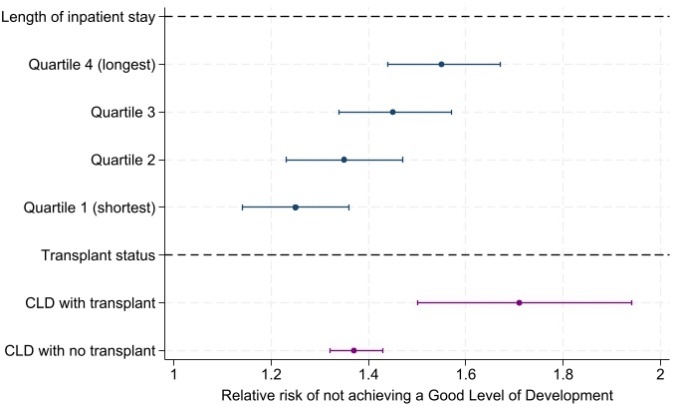
Outcomes by severity: adjusted relative risk of not achieving a good level of development comparing children with versus without CLD by age 5 (academic years 2011/2012–2016/2017). Blue=quartiles of length of hospital stay. Quartile 1=1–3 days of inpatient stay with CLD diagnosis; quartile 2=4–10 days; quartile 3=11–35 days and quartile 4=36+ days. Red=transplant status. CLD, chronic liver disease.

Associations were consistent across all severity measures: effects were stronger for children with a liver transplant compared with those without a transplant and for those who had a longer inpatient stay (36+ days vs 1–3, 4–10 or 11–35 days; figure 2 and online supplemental table S6).

**Figure 3:**
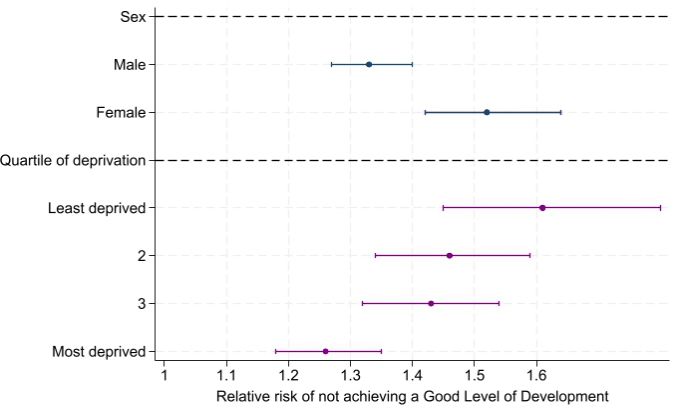
Outcomes by sex and deprivation: adjusted relative risk of not achieving a good level of development comparing children with and without chronic liver disease by age 5 (academic years 2011/2012–2016/2017). Quartile of deprivation is defined using the Income Deprivation Affecting Children Index, an area-based indicator of socioeconomic status in the areas where pupils were living during the Early Years Foundation Stage Profile period.

We found significant interactions by child sex and deprivation (figure 3). The effect of having a CLD diagnosis on the risk of not achieving a good level of development was greater in girls (RR comparing CLD to no CLD 1.52, 95% CI 1.42 to 1.64) than in boys (RR 1.33, 95% CI 1.27 to 1.40). There was also a clear pattern by deprivation, where the effect of having a CLD diagnosis was stronger in those living in less deprived areas.

## Discussion and conclusion

### Main findings

Our study of over 5 million children in England shows that children diagnosed with early-onset CLD are at a substantially increased risk of not achieving a good level of development at school age, with deficits in all areas of development, compared with those without CLD. The good level of development is a key indicator of school readiness, and these results indicate that children with CLD could potentially benefit from further support to reduce these disparities.

A major strength of our study is the use of an official educational assessment in a population-based cohort constructed from novel linked health-education administrative data, capturing a high proportion of the population of children in England. This study, therefore, adds to previous evidence on the developmental delay of children with CLD, restricted by small sample sizes and the applied design and methods of the studies.

A systematic review including 25 studies (1913 children) found deficits in all studies in one or more areas of development, including cognitive, motor and behaviour.^4^ While confirming global developmental delay in children with CLD, we found effect sizes to be most pronounced in the physical domain. In BA, both gross and fine motor skills have been shown to be inferior to norm scores, and although motor development initially improves following liver transplant, these improvements do not persist long term. For children requiring liver transplantation, impaired nutrition peri-transplant, shorter disease duration before transplant and older age at liver transplant were identified as risk factors related to motor delay. However, in 41 infants with BA, a study observed atypical general movements in 46% as early as the time of diagnosis and a median age of 48 days.^14^ Similarly, from an observational study of 50 infants, with BA aged 6 months to 4 years, developmental scores were significantly lower compared with age-controlled infants, with no difference for those who underwent liver transplantation.^15^ These data suggest that the coincidence of early-onset liver disease with a period of rapid brain development is likely to influence neurodevelopmental outcomes.

While the majority of our study population was diagnosed before the age of 1 year, further analysis did identify transplant status and longer length of stay in hospital as risk factors of not achieving a ‘good level of development’ aged 5 years. This is in keeping with previous reports where more days of hospital admission in the first year after liver transplantation and a number of medical complications were associated with impaired cognition before and after liver transplant.^4^

Information on school performance in children with liver disease is limited to post-liver transplantation and questionnaire-based. In 9 out of 18 studies analysed, 42% of children had special educational needs, and between 6% and 55% had to repeat at least one class at school.^4^ The presence of pretransplant special educational needs was found to be a risk factor, while calcineurin inhibitors, the main immunosuppressive agent used after liver transplantation, were associated with both special educational needs and impaired cognition.

The clinical and sociodemographic information captured in ECHILD allowed us to make adjusted comparisons in order to isolate the association between CLD and child development. Despite girls and those living in less deprived areas tending to be more likely to achieve a good level of development in the general population, a novel observation was that girls and those living in less deprived areas were not only over-represented in the group of children with CLD, but were also more likely to experience a disadvantage due to CLD. Gender difference has not been observed in the context of neurodevelopmental outcomes in children with CLD previously, though most studies have focused on children with BA or post-liver transplantation, which comprised a minority of the current study population.^16^ A previous study has observed that adolescent females were as likely as males to screen positive for neurodevelopmental difficulties such as attention-deficit hyperactivity disorder and autism spectrum disorder, which are typically more prevalent in males.^17^ These interaction effects require further exploration.

A study found that living in a one-parent household was linked with lower full intelligent quotient scores in a cohort of 144 children post liver transplantation, which could not be confirmed in other studies to date.^18^ A limitation of our study was that we did not have data on parental education or income.

Overall, liver disease is estimated to affect between 2 and 10/10 000 children when excluding community-acquired acute infectious hepatitis, with the majority of those presenting during infancy expected to develop chronic liver disease.^19 20^ However, this estimate is historical with no accurate current data available. Our data suggests an incidence of 2/1000 children who were diagnosed with CLD before the age of 5 years, in keeping with 1/500 live births. We defined chronic liver disease relying on existing hospital codes (ICD-10): the limitation is that this classification may be subject to error, as we relied solely on hospital records. If a child with CLD did not present to the hospital, if their diagnosis was not recorded, or if we did not include their diagnosis in our code list, they would have been incorrectly classified as not having CLD. Considering that the care for children with liver disease and liver transplantation is centralised in three supraregional centres in the UK, this is unlikely. The incidence of BA of 1/15 000 live births is in keeping with published UK data, suggesting that at least for this well-characterised condition, our approach is robust.^2^

There was some missing data in clinical records (particularly in gestational age), which resulted in a reduction in the sample size. This could result in bias in the association between CLD and child development if missing data are not completely at random. We also only included children who were born in England (in order to be able to capture data on gestational age and size at birth), and so results may not be generalisable to children born in other countries. Previous studies have shown that HES birth records are broadly representative of the wider population of births in England.^21 22^

Our data agree with the observation that within the childhood liver disease cohort, those who have undergone liver transplantation and spent more time in the hospital are more at risk of worse developmental outcomes. The suggestion that females and those in less deprived areas are at higher risk needs further exploration.

In addition, despite its limitations, this study suggests that the frequency of childhood liver disease in children is higher than previously reported; this will need confirmation in other countries. Finally, the current data lends itself to further exploration of the longitudinal educational outcomes of children with CLD, including the different trajectories for those with and without liver transplantation.

## Supplementary material

10.1136/archdischild-2025-329409 online supplemental file 1

## Data Availability

Data may be obtained from a third party and are not publicly available.
