# Supplementary material for: School readiness in children with early-onset chronic liver disease: a population-based linked data cohort in England
Source: Arch Dis Child. 2026 Feb 16;111(6):e329409. doi: 10.1136/archdischild-2025-329409 (PMC13217031; doi:10.1136/archdischild-2025-329409)
Supplement: online supplemental file 1 [file archdischild-111-6-s001.docx]

## Supplementary material

**Table S1.** ICD-10 code list for identifying Chronic Liver Disease in children by age 5

**Table S2.** Differences in standardised scores in the Early Years Foundation Stage Profile according to chronic liver disease by age 5 and child characteristics

**Table S3.** Outcomes across developmental areas at the Early Years Foundation Stage Profile, by exposure to Chronic Liver Disease by age 5 in children attending school in England

**Table S4.** Outcomes by severity: Crude and Adjusted Relative Risk of not achieving a Good Level of Development comparing children with and without Chronic Liver Disease by age 5 (academic years 2011/12-2016/17)

**Table S5.** Outcomes across developmental areas at the Early Years Foundation Stage Profile, by exposure to Chronic Liver Disease by age 5 in children attending school in England

**Table S6.** Outcomes by severity^a^: Crude and Adjusted Relative Risk of not achieving a Good Level of Development comparing children with and without Chronic Liver Disease by age 5 (academic years 2011/12-2016/17)

**Figure S1.** Flowchart Illustrating the Formation of the Study Cohorts

This supplementary material has been provided by the authors to give readers additional information about their work.

**Table S1.** ICD-10 code list for identifying early-onset Chronic Liver Disease in children (diagnosed by age 5)

| **ICD-10 code** | **Description** |
| --- | --- |
| B169 | Acute hepatitis B without delta-agent and without hepatic coma |
| B172 | Acute hepatitis E |
| B178 | Other specified acute viral hepatitis |
| B179 | Acute viral hepatitis, unspecified |
| B181 | Chronic viral hepatitis B without delta-agent |
| B182 | Chronic viral hepatitis C |
| E830 | Disorders of copper metabolism |
| E848 | Cystic fibrosis with other manifestations |
| E880 | Disorders of plasma-protein metabolism, not elsewhere classified |
| K720 | Acute and subacute hepatic failure |
| K729 | Hepatic failure, unspecified |
| K740 | Hepatic fibrosis |
| K743 | Primary biliary cirrhosis/ AISC overlap |
| K744 | Secondary biliary cirrhosis |
| K745 | Biliary cirrhosis, unspecified |
| K746 | Other and unspecified cirrhosis of liver/ cryptogenic cirrhosis |
| K746 | Other and unspecified cirrhosis of liver |
| K754 | Autoimmune hepatitis |
| K758 | Other specified inflammatory liver diseases |
| K760 | Fatty (change of) liver, not elsewhere classified |
| K768 | Other specified diseases of liver |
| K769 | Liver disease, unspecified |
| K830 | Cholangitis |
| P592^a^ | Neonatal jaundice from other and unspecified hepatocellular damage |
| P593^a^ | Neonatal cholestasis |
| Q442 | Atresia of bile ducts |
| Q446 | Cystic disease of liver/ CHF |
| Q447 | Other congenital malformations of the liver |

ICD-10 = International Classification of Diseases, Tenth Revision

^a^ Children who only presented with jaundice before 12 months but had no other CLD pathology by age 5 were not classified as having CLD

**Table S2.** Operating Procedure Codes Supplement 4 (OPCS-4) code list for identifying liver transplant in children by age 5.

| **Code** | **OPCS-4 description** |
| --- | --- |
| J01.1 | Orthotopic transplantation of liver NEC |
| J01.2 | Heterotopic transplantation of liver |
| J01.3 | Replacement of previous liver transplant |
| J01.4 | Transplantation of liver cells |
| J01.5 | Orthotopic transplantation of whole liver |
| J01.8 | Other specified transplantation of liver |
| J01.9 | Unspecified transplantation of liver |

**Table S3.** Age at diagnosis for Chronic Liver Disease

| **Age at diagnosis (years)** | **N** | **%** |
| --- | --- | --- |
| 0 | 2,123 | 57.8 |
| 1 | 395 | 10.8 |
| 2 | 331 | 9.0 |
| 3 | 243 | 6.6 |
| 4 | 283 | 7.7 |
| 5 | 297 | 8.1 |
| *Total* | 3,672 |  |

**Table S4.** Differences in standardised scores in the Early Years Foundation Stage Profile according to chronic liver disease by age 5 and child characteristics

|  | **2007/08-2010/11**  N = 1,197,873 | **2011/12-2016/17**  N = 2,297,765 |
| --- | --- | --- |
| *Crude model* |  |  |
| **Chronic Liver Disease by age 5** | -0.81 (-0.89, -0.74) | -0.53 (-0.58, -0.49) |
| *Fully Adjusted model* |  |  |
| **Chronic Liver Disease by age 5** | -0.69 (-0.81, -0.58) | -0.41 (-0.46, -0.36) |
| **Sex** |  |  |
| Female | Ref. | Ref. |
| Male | -0.31 (-0.31, -0.3) | -0.34 (-0.34, -0.33) |
| **Ethnicity** |  |  |
| White | Ref. | Ref. |
| Asian | -0.09 (-0.1, -0.08) | -0.08 (-0.09, -0.08) |
| Black | -0.04 (-0.05, -0.03) | 0.00 (-0.01, 0.00) |
| Chinese | 0.00 (-0.03, 0.03) | -0.05 (-0.07, -0.03) |
| Any other ethnic group | -0.12 (-0.14, -0.11) | -0.15 (-0.16, -0.14) |
| Mixed | 0.07 (0.06, 0.08) | 0.07 (0.07, 0.08) |
| **IDACI Score (quartile)** | -0.95 (-0.96, -0.94) | -0.93 (-0.94, -0.92) |
| 1^st^ (More deprived) | Ref. | Ref. |
| 2^nd^ | -0.16 (-0.17, -0.16) | -0.14 (-0.15, -0.14) |
| 3^rd^ | -0.33 (-0.34, -0.33) | -0.30 (-0.30, -0.29) |
| 4^th^ (Less deprived) | -0.49 (-0.49, -0.48) | -0.43 (-0.43, -0.43) |
| **Gestational age (weeks)** |  |  |
| 24-31 | -0.59 (-0.61, -0.57) | -0.59 (-0.61, -0.57) |
| 32-33 | -0.32 (-0.34, -0.30) | -0.32 (-0.34, -0.30) |
| 34-36 | -0.21 (-0.22, -0.20) | -0.21 (-0.22, -0.20) |
| 37-38 | -0.11 (-0.12, -0.11) | -0.11 (-0.12, -0.11) |
| 39 | -0.04 (-0.04, -0.03) | -0.04 (-0.04, -0.03) |
| 40 | Ref. | Ref. |
| 41-43 | 0.01 (0.01, 0.02) | 0.01 (0.01, 0.02) |
| **Size for gestation** |  |  |
| Small (<10 centile) | -0.18 (-0.18, -0.17) | -0.18 (-0.18, -0.17) |
| Normal | Ref. | Ref. |
| Large (>90 centile) | 0.04 (0.04, 0.05) | 0.04 (0.04, 0.05) |
| **Maternal age (years)** |  |  |
| <20 | -0.37 (-0.37, -0.36) | -0.37 (-0.37, -0.36) |
| 20-24 | -0.24 (-0.25, -0.24) | -0.24 (-0.25, -0.24) |
| 25-30 | -0.09 (-0.10, -0.09) | -0.09 (-0.10, -0.09) |
| 30-34 | Ref. | Ref. |
| 35-39 | -0.02 (-0.02, -0.01) | -0.02 (-0.02, -0.01) |
| 40-65 | -0.08 (-0.09, -0.07) | -0.08 (-0.09, -0.07) |

IDACI = Income deprivation affecting children index

**Table S5.** Outcomes across developmental areas at the Early Years Foundation Stage Profile, by exposure to Chronic Liver Disease by age 5 in children attending school in England

|  |  | **No chronic liver disease** | | **Chronic liver disease** | |
| --- | --- | --- | --- | --- | --- |
|  |  | No. | % | No. | % |
| **Achieved a Good Level of Development 2011/12-2016/17** | |  |  |  |  |
|  | Yes | 1,780,048 | 64.7 | 993 | 44.2 |
|  | No | 972,102 | 35.3 | 1,256 | 55.8 |
|  | | Mean | SD | Mean | SD |
| **Developmental areas for 2007/08-2010/11** | |  |  |  |  |
|  | Total EYFSP score | 0.00 | 1.00 | -0.87 | 1.65 |
|  | Personal, Social and Emotional Development | 0.00 | 1.00 | -0.73 | 1.59 |
|  | Communication, Language and Literacy | 0.00 | 1.00 | -0.79 | 1.48 |
|  | Problem Solving, Reasoning and Numeracy | 0.00 | 1.00 | -0.83 | 1.63 |
| **Developmental areas for 2011/12-2016/17** | |  |  |  |  |
|  | Total EYFSP score | 0.00 | 1.00 | -0.58 | 1.17 |
|  | Communication and Language | 0.00 | 1.00 | -0.50 | 1.11 |
|  | Physical Development | 0.00 | 1.00 | -0.58 | 1.19 |
|  | Personal, Social and Emotional Development | 0.00 | 1.00 | -0.50 | 1.15 |
|  | Literacy | 0.00 | 1.00 | -0.43 | 1.03 |
|  | Understanding the World | 0.00 | 1.00 | -0.54 | 1.19 |
|  | Mathematics | 0.00 | 1.00 | -0.47 | 1.06 |
|  | Expressive arts, designing and making | 0.00 | 1.00 | -0.49 | 1.15 |

Abbreviations: IDACI, Income deprivation affecting children index; SD: Standard Deviation; EYFSP, Early Years Foundation Stage Profile.

**Table S6.** Outcomes by severity of disease: Adjusted Relative Risk of not achieving a Good Level of Development comparing children with and without Chronic Liver Disease by age 5 (academic years 2011/12-2016/17)

|  |  | **Adjusted relative risk (95% CI)** |
| --- | --- | --- |
| **No chronic liver disease** |  | Ref. |
| **Chronic liver disease** | Length of inpatient hospital stay^a^ = 1-3 days | 1.25 (1.14, 1.36) |
|  | Length of inpatient hospital stay^a^ = 4-10 days | 1.35 (1.23, 1.47) |
|  | Length of inpatient hospital stay^a^ = 11-35 days | 1.45 (1.34, 1.57) |
|  | Length of inpatient hospital stay^a^ = 36+ days | 1.55 (1.44, 1.67) |
| **No chronic liver disease** |  | Ref. |
| **Chronic liver disease** | No transplant | 1.37 (1.32, 1.43) |
|  | Transplant | 1.71 (1.50, 1.94) |

^a^ Admissions with a diagnosis of chronic liver disease

**Figure S1.** Flowchart Illustrating the Formation of the Study Cohorts

2.360 records excluded due to duplication in NPD ID

995 excluded due to missing on EYFSP standardised total point score in academic years 2007/08-2010/11

292 missing data in PSE 07/11

88 missing data in PSE 07/11

199 missing data in PSRN 07/11

3545 excluded due to missing on EYFSP standardised total point score in academic years 2012/13-2016/17

Pupils with Early Years Foundation Stage (EYFS) Profile in academic years 2007/08 to 2016/17 (with a record in HES) born (in England) between 1 September 2000 and 31 August 2012.

N=5,092,150

Pupils included in the Analysis before exclusions for missing data in Covariates.

N=5,084,671

N= 2,330,272 (academic years 2007/08-2010/11)

N= 2,754,399 (academic years 2011/12-2016/17)
